# Complex Crystallization Kinetics of a Mg–Al Hydrotalcite and Their Practical Implications from the Process Point of View

**DOI:** 10.1021/acs.iecr.1c01785

**Published:** 2021-08-02

**Authors:** Marco-Antonio López-Martínez, Ignacio Melián-Cabrera

**Affiliations:** † División de Ciencias Básicas e Ingeniería, Universidad Autónoma Metropolitana-Unidad Azcapotzalco, Av. San Pablo 180, Col. Reynosa Tamaulipas, Alc. Azcapotzalco, 02200 Mexico City, Mexico; ‡ Applied Photochemistry and Materials for Energy Group, University of La Laguna, Avda. Astrofísico Francisco Sánchez, s/n, P.O. Box 456, 38200 San Cristóbal de La Laguna, Santa Cruz de Tenerife, Spain

## Abstract

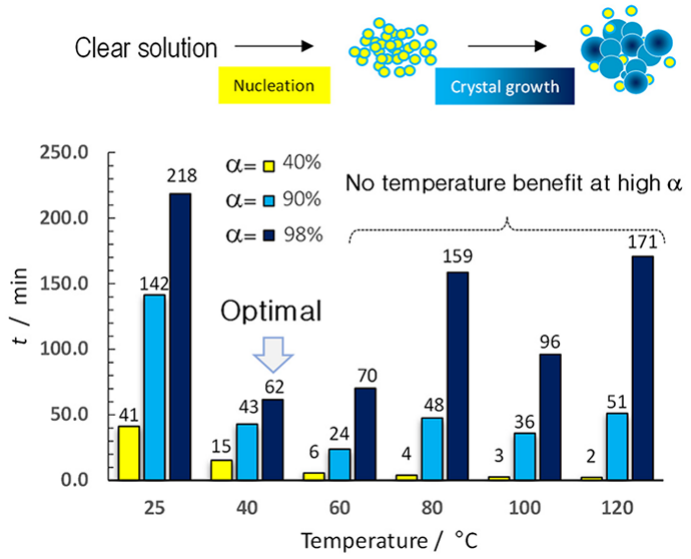

Hydrotalcites are an important class of layered materials, displaying ion-exchange, adsorption, and base catalytic properties. The crystallization kinetics for hydrotalcites are however hardly available. Nevertheless, as their reconstruction from the oxides (also called as “memory effect”) is considered a synthesis route, this can be used to study the crystallization phenomena. This note looks at the reconstruction kinetics of a Mg–Al hydrotalcite using previously reported kinetic expressions. It was found that high temperature is beneficial if the process is controlled by nucleation. The temperature effect is less obvious when the process reaches a diffusional control regime. For example, temperature is beneficial to shorten the processing time in a nucleation-regime conversion (e.g., 40%). However, to achieve a high conversion (e.g., 98–99%), an intermediate temperature shows the optimal condition, i.e., shortest processing time. The work shows useful side effects of diffusional control. It also highlights the importance of obtaining the kinetics over the entire range for process optimization and, finally, emphasizes that both parameters in the Avrami–Erofe’ev model impact the time required to achieve a given conversion. Directions for further studies to understand the kinetic-process relationships have been highlighted.

Hydrotalcites are layered materials that find applications in the pharmaceutical industry, plastic processing as a stabilizer, catalysis, ion exchange, and adsorption.^1−5^ In heterogeneous catalysis, hydrotalcites or activated hydrotalcites are effective in reactions such as aldol condensation,^6,7^ Claisen–Schmidt condensation,^8^ and glycerol carbonate synthesis,^9,10^ to cite a few. They have also evidenced potential for CO_2_ adsorption from flue gases.^4,11,12^ As an ion-exchanger, these materials are very effective for removing undesired anions, such as NO_3_^–^, from wastewater^13,14^ to avoid eutrophication. Their general formula corresponds to a layered double hydroxide, given as [M^2+^_1–*x*_M^3+^_*x*_(OH)_2_]^*x*+^[A^*n*−^_*x*/*n*_]^*x*−^·*m*H_2_O, where M^2+^ is a divalent cation such as Mg^2+^, Ca^2+^, etc.; M^3+^ is a trivalent cation such as Al^3+^, Cr^3+^, etc.; and A^*n*–^ is the anion, typically CO_3_^2–^, though it can host many types such as NO_3_^–^, Cl^–^, ClO_4_^–^, etc.^15,16^ The hydrated anion is in the interlayer space. Therefore, these groups can be exchanged by other anionic species.

The common methodology for preparing hydrotalcites is via wet chemical synthesis by coprecipitation.^15−17^ The metal cations are precipitated with an alkaline solution at a fixed temperature and pH. It is well-known that the product crystallinity is affected by many synthesis variables; hence, it is important to control them carefully to obtain reproducible materials.^18−20^ In particular, de Roy et al.^20^ provide many syntheses and postsynthesis cases, showing these effects. A more recent example can be mentioned about synthesis conditions resulting in new porosities, the formation of a hierarchical mesoporous and macroporous Al–Mg hydrotalcite.^21^

After the synthesis, the material is often thermally activated at moderate temperatures, 300–500 °C. This process has been extensively studied, and few models have been proposed.^22−24^ On some occasions, the synthesis of a hydrotalcite is not aiming at a base catalyst but to achieve a high metal interdispersion that cannot be obtained by other methods. In that situation, a hydrotalcite provides an optimal metal oxide interdispersion after calcination, leading to minimal sintering during the thermal activation. This is for instance the case of the Cu–Zn–Al mixed oxide catalyst.^25−27^

The product derived from hydrotalcites after calcination shows a phenomenon that has been denoted as a “memory effect” (other terms for this phenomenon is rehydration, reconstruction, and reverse topotactic transformation). In this process, the calcined product returns to the original hydrotalcite. Studies on this effect have been carried out in the liquid phase under various alkaline conditions, such as Na_2_CO_3_,^28,29^ K_2_CO_3_^30^ KOH,^30^ or diluted solutions containing various types of anions with ion-exchange behavior (SO_4_^2–^, F^–^, HPO_4_^2–^, Cl^–^, B(OH)_4_^–^, and NO_3_^–^).^31^ The effect is also seen when the calcined material is treated in liquid water^17,32−35^ or in wet gas streams.^35−37^ These latter rehydration steps opened new synthetic routes to produce hydrotalcite-based heterogeneous catalysts with tuned basicity.^38^ Takehira^19^ provided an exhaustive review in this direction. Two mechanisms to explain the reconstruction effect have been proposed. The first one proposed by Sato et al.^32^ refers to a reverse topotactic transformation, where the material is reconstituted back to the original structure without dissolution. In a second interpretation,^29,34^ the material would be dissolved and subsequently recrystallized.

A conventional direct synthesis embraces the aqueous coprecipitation of soluble metal cations (e.g., nitrates, sulfates) with a precipitating agent (e.g., Na_2_CO_3_, NaOH, urea), whereas the so-called “memory effect” initiates with the calcined hydrotalcite (i.e., a solid) in the presence of a base solution or water/steam. Recently, Mascolo and Mascolo^39^ appraised the “memory effect” concept and arrived to the conclusion that it has been improperly defined. Most of the reported “memory effect” recipes are based on a “direct synthesis” approach involving poorly crystalline and, therefore, highly reactive oxides. These oxides in the presence of liquid/gas water react to produce a meixnerite structure (or a hydrotalcite structure in the presence of a solution containing anions). The authors made an exception: the removal of the interlayer water of a hydrotalcite rendering a highly disordered structure. The recovery of the hydrotalcite is considered to be a true memory effect. The important aspect here is that the phenomenon of the dissolution/recrystallization may be indeed a “direct synthesis” as suggested by Mascolo–Mascolo’s appraisal.^39^

Despite hydrotalcites being highly relevant materials, their crystallization kinetics have been hardly reported to the best of our knowledge. Having a kinetic expression would however be very relevant to design the synthesis reactor and to optimize the process conditions. A possible approach to study their crystallization is considering the Mascolo–Mascolo’s principle,^39^ i.e., most of the reconstruction processes are a direct synthesis, since those oxides are highly reactive species. Based on that, the work of Millange et al.^29^ (kinetics of the reconstruction) can be used for further analysis. They quantified the *in situ* reconstruction of a calcined Mg–Al hydrotalcite, i.e., starting with a highly dispersed MgO/Al_2_O_3_ oxide in an aqueous sodium carbonate solution, by energy dispersive X-ray diffraction using white-beam synchrotron-generated X-rays. They found that the reconstruction follows the Avrami–Erofe’ev nucleation–growth model.^40,41^ In this note, the kinetic constants proposed by Millange et al.^29^ are further analyzed and practical conclusions are proposed.

The authors tried to adjust the crystallization curves by least-squares refinement, but it did not work. At intermediate temperatures, it was impossible to obtain a good fitting to the entire data set using a single model equation. They observed that *n*-values varied with temperature, evidencing that the mechanism is different with temperature changes. The *k*-values showed no obvious pattern with temperature, and the growth curves had a different shape, which indicated the change of the reaction mechanism with temperature. To clarify the matter, the authors applied the Sharp–Hancock’s linearization^42^ that allowed detecting two distinctive reaction steps, with a characteristic *n*-value for each regime. The first step had a positive trend of the *k*-value with temperature, whereas the second step did not. The second step, having an *n* around 0.5 was ascribed to a diffusion-limited process. That is, the rate of the reaction depends only on the diffusion of the reactive species through the solution into the crystallization site and not to the nucleation rate.

We took the set of kinetic constants for both steps and made the Arrhenius plots (Figure 1). For the second step (Figure 1B), it clearly shows a transition from a chemically controlled process to a film diffusion-limited one, since the activation energy changed from 66 kJ/mol to nil; the latter value is characteristic of film-diffusion control (i.e., external mass transfer) since the mass transfer coefficient has a weak dependency with temperature.^43−45^ In fact, a nil value means that a strong limitation takes place. Moreover, the first step also shows a decay of the activation energy from 51 to 14 kJ/mol (Figure 1A). The latter value indicates that mass transfer also plays a role at low conversion in the nucleation step. A value of 14 kJ/mol is low to be considered as an internal mass transfer-limited step; considering 51 kJ/mol is the intrinsic activation energy, the internal mass transfer activation energy should be half of that, i.e., ∼26 kJ/mol.^43−45^ Therefore, a value of 14 kJ/mol can be ascribed to a transition from internal mass transfer into a film-diffusion control regime. Figure 1C summarizes the two regimes visually with a model, as nucleation and crystal growth, with the proposed kinetic features.

**Figure 1:**
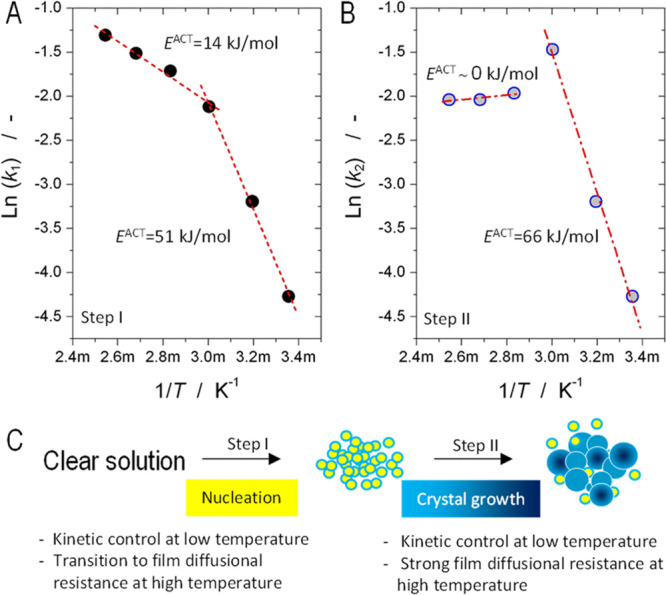
Arrhenius plots for Step I (A) and Step II (B). Data at 25 and 40 °C are shared for both steps. (C) Model representing the hydrotalcite synthesis, including nucleation (Step I) and crystal-growth regimes (Step II).

Having a low apparent activation energy means that temperature has little influence on the kinetics. It is reasonable to consider mass transfer limitations based on the kinetic theory for heterogeneous systems. However, heat transfer limitations should not be ruled out entirely. A possible explanation is that at high temperature, the formed crystallites are surrounded, partly or temporarily, by gas bubbles that inhibit the mass and heat transfer, therefore inhibiting the crystal growth. Based on these observations, we looked at the practical implications from a process point of view.

Working with aqueous solutions at the industrial scale and, in particular, heating these liquids or slurries can be energy consuming due to the high heat capacity of water. This can be seen in Figure S-1, where the energy consumption to heat a slurry containing 1 tonne of oxide at the conditions reported by Millange at al.^29^ has been calculated; the accompanying water is 7.246 tonnes. It can be seen that the scale is very high, reaching ∼3 GJ at 120 °C, due to the high heat capacity of water. It can also be seen that heating the solid has a minor impact (difference between both lines), due to the lower heat capacity of the solid. Therefore, based on these high energy figures, we looked at the temperature effect on the crystallization to understand what the optimal temperature is and to reduce heating costs. For this, we first represented the crystallization curves according to Millange’s model, having two steps. This can be found in Figure 2. The crystallization curves were constructed with both steps for temperatures ≥60 °C. For temperatures below 60 °C, a single-step model was proposed. The graphs evidence that the diffusional control (Step II) becomes dominant with the increasing temperature, whereas the nucleation (Step I) becomes shorter. That means that at higher temperatures, the nucleation kinetic is faster and the process becomes mass-transport limited earlier in time. To understand the effect of temperature on both steps, the curves were comparatively plotted in Figure 3, for Step I (Figure 3A) and Step II (Figure 3B). The kinetics for Step I shows a conventional trend; the conversion curves move to the left, meaning that at equal reaction time, the conversion is higher at higher temperature. This can be visualized with the dashed arrow, for a generic reaction time. The kinetics for Step II shows a complex behavior. Between 25 and 60 °C, the trend is positive with respect to temperature. At higher temperatures, the conversion curves decay. Those trends can be seen with the dashed lines for a generic reaction time. That means that from a practical point of view, increasing the temperature to accelerate the reaction rate, in order to achieve high conversions with reduced time, does not help.

**Figure 2:**
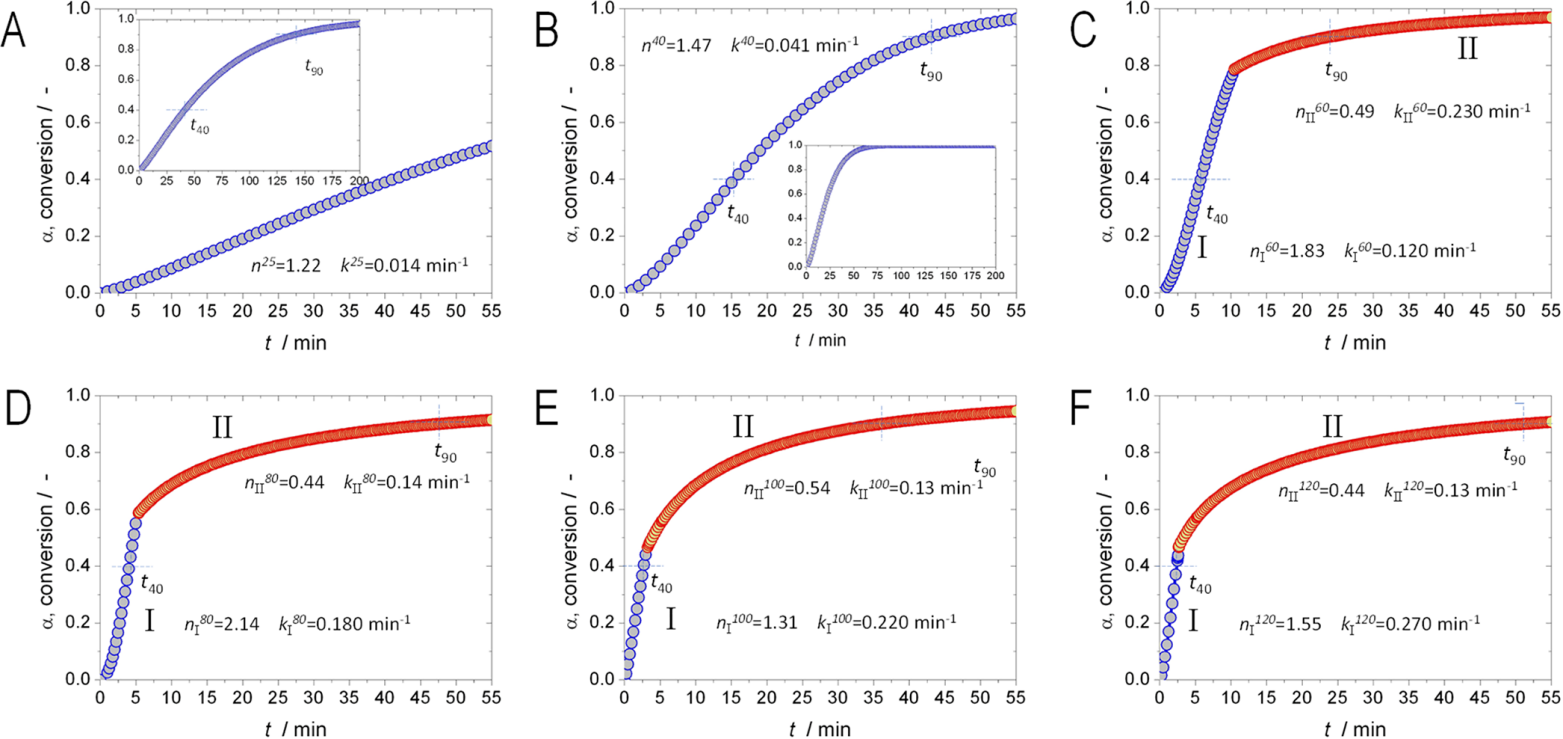
Constructed crystallization curves for a Mg–Al hydrotalcite using tabulated kinetic data (*k* and *n* values) as reported by Millange at al.^29^ at (A) 25 °C, (B) 40 °C, (C) 60 °C, (D) 80 °C, (E) 100 °C, and (F) 120 °C. The terms *t*_40_ and *t*_90_ correspond to the time required to achieve 40 and 90% conversion, respectively. The values of the kinetic constants (*k* and *n*) are included for clarity.

**Figure 3:**
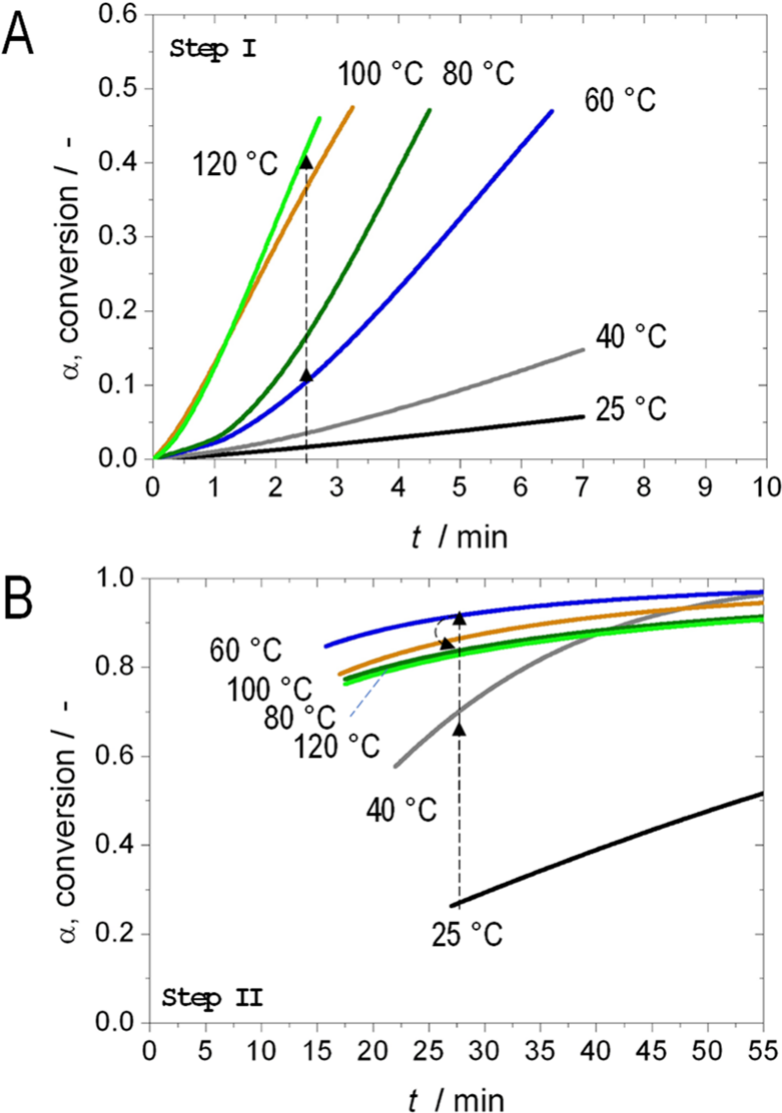
Effect of temperature on conversion (α) for both processes; Step I (A) and Step II (B).

This effect can be explained in a different manner by calculating the time required to achieve a certain conversion. This parameter can provide a more practical estimate. The reader is referred to the Methods section for more information about the employed equations. Figure 4A represents the time to achieve a conversion in both regimes, as an example, 40% in Step I and 90% in Step II. These parameters can be seen in Figure 2, where *t*_40_ and *t*_90_ have been indicated. The representation of *t*_40_ (Figure 4A) indicates that the time required to achieve 40% conversion decreases with the temperature. This is due to the positive Arrhenius trend of *k*_1_ with temperature (and relatively constant *n*_1_-values). The trend for *t*_90_ is also decreasing with temperature, but it then increases at 80 °C and remains high at higher temperatures. This is ascribed to the anomalous trends of the *k*_2_, with similar *n*_2_-values. This plot clearly shows that the optimal temperature to achieve 90% conversion is 60 °C and not a higher value.

**Figure 4:**
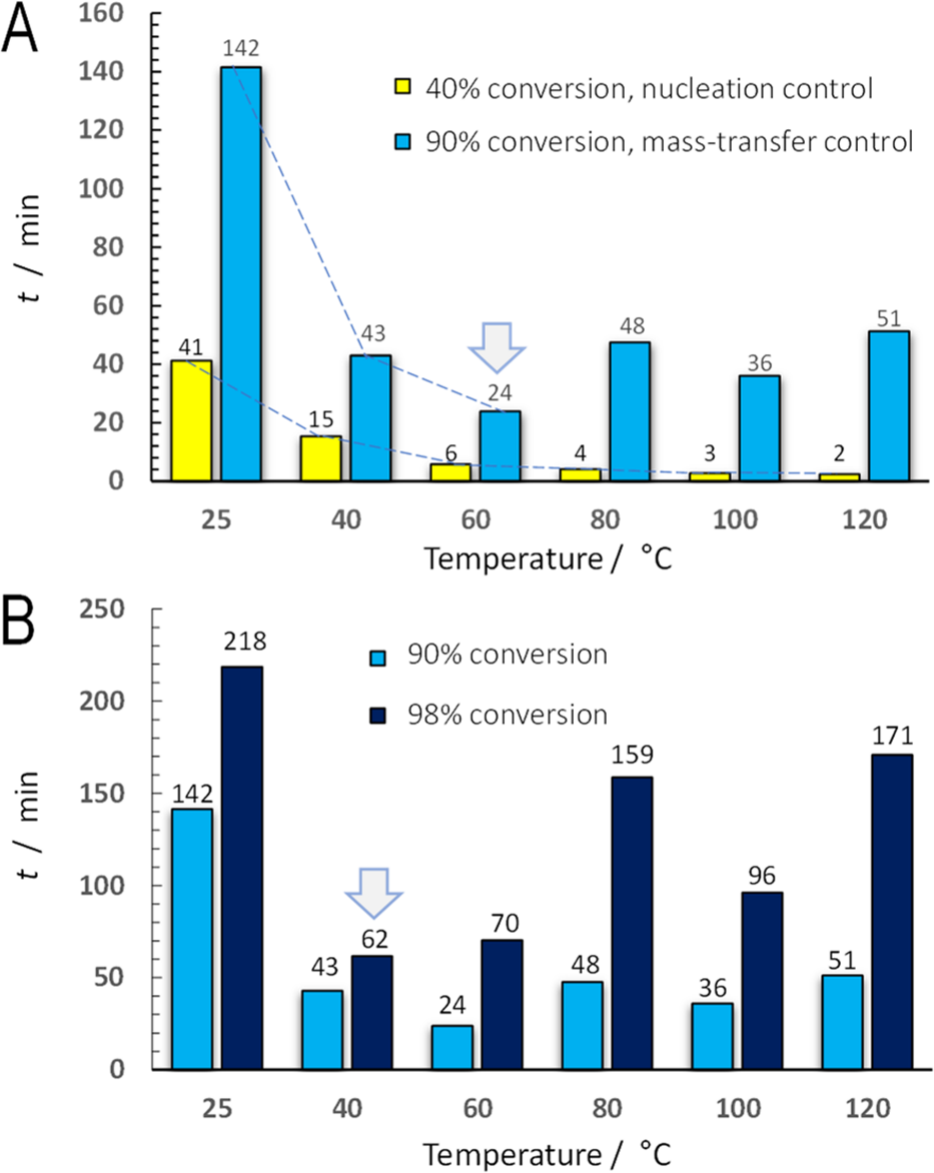
A) Effect of temperature on reconstruction time to achieve conversion levels of 40 and 90%. (B) Effect of temperature on reconstruction time to achieve conversion levels of 90 and 98%.

The Avrami–Erofe’ev’s model predicts that 100% conversion would require an infinite reaction time, as it progresses asymptotically to 100% with time. However, we can consider 98% as a practical conversion to achieve a completely processed batch in practice. The time to achieve 98% conversion (*t*_98_) was plotted in Figure 4B, together with *t*_90_ as a point of comparison. It is interesting to note that the optimal temperature (i.e., shorter processing time to achieve 98% conversion) is 40 °C, rather than 60 °C which was found optimal at 90%, whereas the kinetic constant at 60 °C (0.23 min^–1^) is higher than that at 40 °C (0.041 min^–1^). This is a biased contradiction as we must also take into account the *n*-values. The *n*-values are different; 1.47 at 40 °C, higher than that at 60 °C with a value of 0.49 (at high conversion). In other words, the *n*-value can also contribute positively in the kinetic rate; therefore, looking only at the *k*-values can lead to wrong conclusions. The same trend is found at 99% conversion, with an optimal temperature of 40 °C (Figure S-2, in the Supporting Information). Figure 5 shows this effect graphically. The conversion is in general higher at 60 °C at equal reaction times. At a certain point, the values at 40 °C overpasses those at 60 °C, which implies that the corresponding processing times are shorter (*t*_98_ and *t*_99_ are highlighted in the figure). This is due to the higher *n*-value at 40 °C.

**Figure 5:**
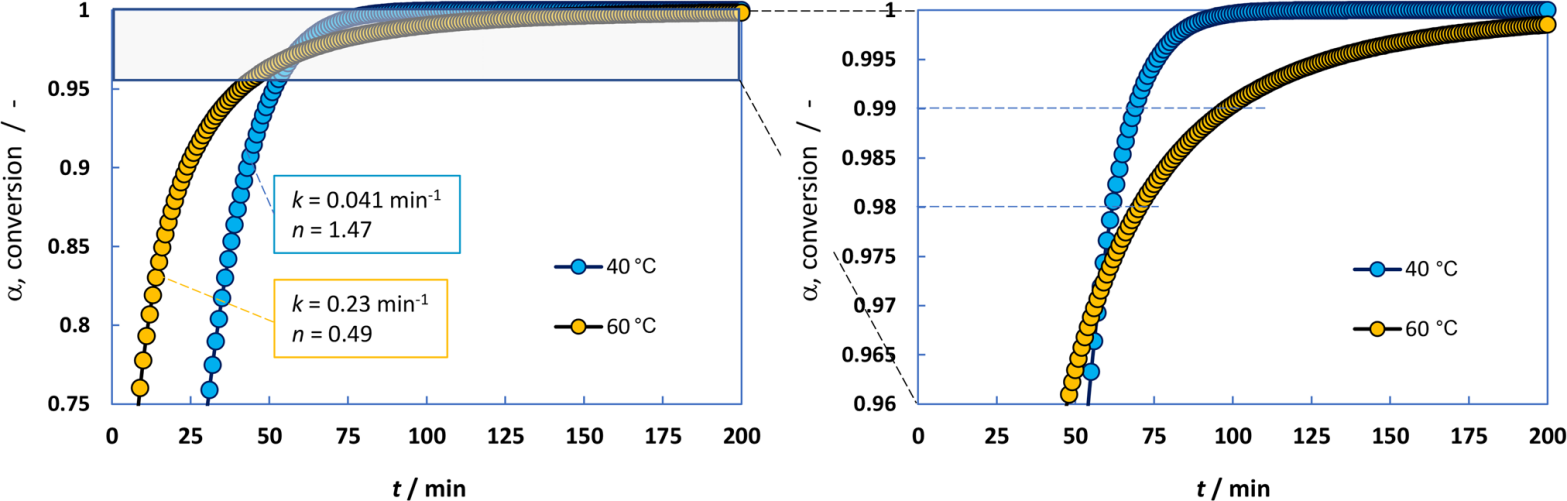
(Left) Constructed crystallization curves at 40 and 60 °C. (Right) Amplification of the region 0.96 to 1 (α).

The above findings provide evidence that high temperatures are not necessary to achieve the optimal synthesis of the Mg–Al hydrotalcite via this reconstruction method. This is very important as energy can be saved from the practical point of view. However, the conclusions from this study cannot be extrapolated to any hydrotalcite; each case must be studied from the kinetic point of view, using the tools described in previous studies^29^ and in this continuing work.

This study shows that crystal growth has a complex effect with regard to temperature. This effect can be due to opposite phenomena taking place, such as heat-conduction-limited growth, diffusion-limited growth, or both simultaneously or even interface source-limited growth. The problem here is complex since the hydrotalcite reconstruction at high temperature involves solid, liquid, and gas phases altogether. Some theoretical frames on isolated cases have been reported,^46^ but the hydrotalcite system has not been, to the best of our knowledge, described using theoretical frames. It is therefore an opportunity to develop a theoretical frame of the temperature effect for the hydrotalcite synthesis, considering more compositions.

Even though the response considered in this study is conversion, the impact of temperature on the final particle size (or particle size distribution) is something still to be understood. Note that particle size is here referred to the crystallite size determined by XRD. Such information was not reported by Millange at al.^29^ They showed in qualitative terms a decay of the full width at half maximum (fwhm) with time and temperature, implying an increase of the particle size with time and temperature (according to the Scherrer equation, which relates the crystallite size with the fwhm).^47^ Their kinetic evaluation was done using the change of the (003) intensity. In theory, when the crystallinity of a material increases, the peak intensity increases while the fwhm decreases. Therefore, the intensity may be used to estimate the particle size, roughly as a first-hand approach. To shed light on this, we used a short-cut method considering the α-curves of our study and some reference values of the particle size reported by Millange et al.^29^ at 100 min of reconstruction. More information about this methodology can be found in the Methods section and Figure S-3. Figure S-3 shows the evolution of the particle size with time at each temperature. The value of the particle size was determined at the time to achieve 99% conversion (Table S-1); such values range between 11.1 and 14.5 nm, for temperatures ranging from 25 up to 120 °C. At 90% conversion (Table S-2), the particle size ranged from 10.1 to 13.2 nm. In both cases, no much variation of the particle size was found. That means that temperature does not seem to have a large impact on the particle size at high conversion levels for this method. In practical terms, operating at lower temperature to save processing time or energy (i.e., 40 °C, optimization discussed earlier) does not sacrifice the particle size. However, this statement should be verified using a more rigorous fwhm approach to make a sound conclusion.

There are additional topics to be considered in future work to enhance the understanding. First, studies to comprehend the effect of mixing on the crystallization, in particular on the mass-transfer limited step, is a possible direction to modify the kinetics and optimize the process. Second, supersaturation can be measured and systematically changed to assess the rate-limiting step. This might provide some understanding of growth kinetics and the reason for high conversion time at high temperature. In fact, high supersaturation favors the nucleation of smaller crystallites, but the reason for the slow growth is not well understood yet. Supersaturation will likely affect the kinetic constants and alter the processing time. Finally, the particle size assessment can be combined with adsorption experiments of the reconstructed products, in comparison with a benchmarked hydrotalcite.

In conclusion, the study shows that, in practical terms, carrying out the crystallization of a hydrotalcite via reconstruction at a moderate temperature is optimal (e.g., 40 °C to achieve 98–99% conversion), as it would require the shortest processing time, while energy for heating up the reaction mixture is saved. This is because higher temperatures do not accelerate the kinetics, likely due to film diffusion-limited mass transfer. Note that this study and its conclusions are based on a previously reported kinetic model done at specific conditions, which requires further expansion to other materials and other synthesis conditions. The study also evidenced the importance of both parameters, *k* and *n*, in the Avrami–Erofe’ev model; the *k*-values cannot be compared alone but in combination with the *n*-values, using for instance a graph representing the conversion, or the time to achieve a certain conversion, in a comparative plot.

## Methods

The Avrami–Erofe’ev model,^40,41^eq 1, consists of the following expression:
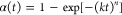
1where *k* (min^–1^) and *n* (−) are the Avrami–Erofe’ev parameters. The Sharp and Hancock^42^ linearization, eq 2, is an alternative mathematical approach to analyze kinetic information. It consists of taking double logarithms of the Avrami–Erofe’ev model:
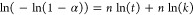
2

The time required to achieve a certain conversion, *t*_α_, can be obtained analytically from eq 3 as
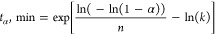
3

The particle size was tentatively estimated using eq 4 as
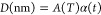
4where α(*t*) is given by eq 1 and *A*(*T*) is a parameter that depends on temperature. It can be calculated when knowing the particle size at one time for each temperature. For this, we employed the average particle size values at 100 min reconstruction reported by Millange et al.^29^ Additional information can be found in Figure S-3.

The heating energy of the solid was calculated as
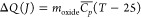
5

The heating energy of the accompanying water was calculated as
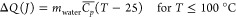
6
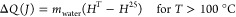
7where *m* is the mass,  is the average heat capacity, *T* is the temperature, and *H* is the corresponding enthalpy at a temperature *T* or 25 °C. This latter temperature was considered as an unheated case, i.e., reference conditions. More information can be found in Figure S-1.

## Supporting Information Available

Figure S-1, energy consumption for heating a slurry containing 1 tonne of calcined hydrotalcite and 7.246 tonnes of accompanying water; Figure S-2, reconstruction time to achieve a conversion level of 99% at various synthesis temperatures; Figure S-3, shortcut method to assess the particle size using the α(*t*)-curves; Table S-1, shortcut method to determine the crystallite size at 99% conversion; Table S-2, shortcut method to determine the crystallite size at 90% conversion; all data refers to a hydrotalcite with a Mg:Al = 3:1 (at.) (PDF)
