# Supplementary material for: Complex Crystallization Kinetics of a Mg–Al Hydrotalcite and Their Practical Implications from the Process Point of View
Source: Ind Eng Chem Res. 2021 Aug 2;60(31):11848–54. doi: 10.1021/acs.iecr.1c01785 (PMC8675131; doi:10.1021/acs.iecr.1c01785)
Supplement: Supplementary file 1 — ie1c01785_si_001.pdf [file ie1c01785_si_001.pdf]

Electronic Supporting Information

A note on the complex crystallization kinetics of a Mg-Al hydrotalcite and their practical implications from the process point of view

Marco-Antonio López-Martínez[a] and Ignacio Melián-Cabrera[b]*

a. División de Ciencias Básicas e Ingeniería, Universidad Autónoma Metropolitana-Unidad Azcapotzalco, Av. San Pablo 180, Col. Reynosa Tamaulipas, Alc. Azcapotzalco 02200, Mexico City, Mexico.

b. Applied Photochemistry and Materials for Energy Group, University of La Laguna, Avda. Astrofísico Francisco Sánchez, s/n, PO BOX 456, 38200 San Cristóbal de La Laguna, S/C de Tenerife, Spain.

Corresponding author: ignacio.melian-cabrera@ull.edu.es

Additional information

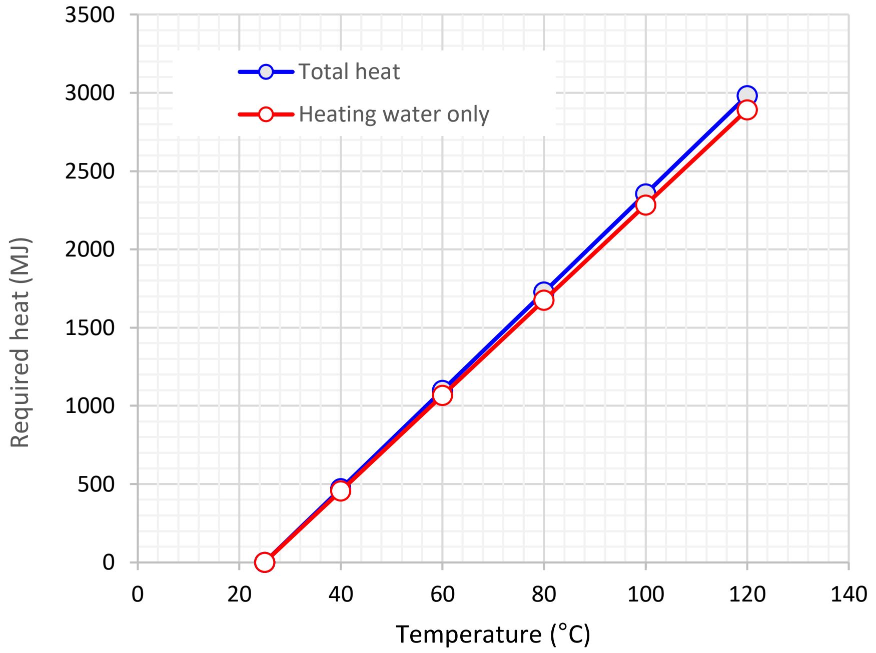

Figure S-1. Energy consumption for heating a slurry containing 1 tonne of calcined hydrotalcite and 7.246 tonnes of water (escalating the conditions employed by Millange et al. *J. Mater. Chem*. **2000**, 10, 1713-1720). The calcined hydrotalcite having a Mg:Al=3 (at.), corresponds to a mixture of ~70 *wt.%* MgO and ~30 *wt.%* Al_2O_3, having an average heat capacity of 954 J/kg/K. This value was calculated from the tabulated data for MgO and Al_2O_3 (Lide, D. R. (Ed.), *CRC Handbook of Chemistry and Physics*, 89th Edition, CRC Press: Boca Raton, 2008, p. 12-200). The average heat capacity for water was taken as 4199 J/kg/K (Lide, D. R. (Ed.), *CRC Handbook of Chemistry and Physics*, 89th Edition, CRC Press: Boca Raton, 2008, p. 6-4). The last point for water at 120 °C was calculated using the enthalpy values since the heat capacity was limited up to 100 °C. The enthalpy values were taken from Borgnakke and Sonntag, *Fundamentals of Thermodynamics*, Wiley: Hoboken, 2014, p. 695. The graph displays the energy for heating the water only and water plus solid (referred as total heat).

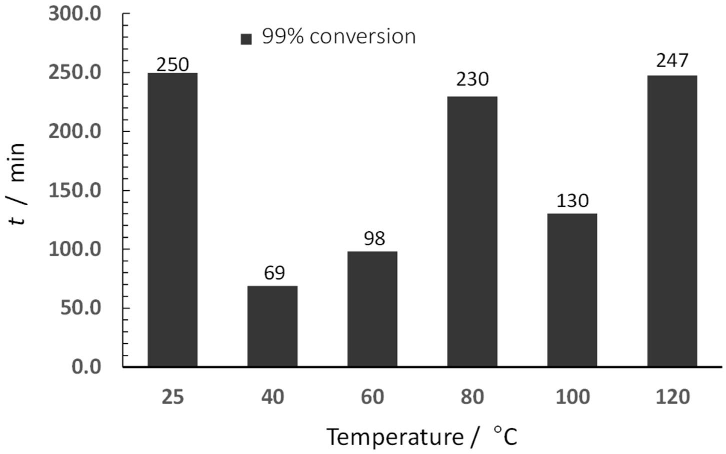

Figure S-2. Reconstruction time (min) to achieve a conversion level of 99% at various synthesis temperatures for a Mg-Al hydrotalcite.

Table S-1. Short-cut method to determine the crystallite size at 99% conversion.

T (°C)	t_{90} (min)	*D* (nm) [a]
25	250	11.4
40	69	11.1
60	98	12.4
80	230	13.7
100	130	13.8
120	247	14.5

a. Using the equation *D* (nm)=*A*(*T*)·α(*t*), where the *A*(*T*) values are given in Figure S-3.

Table S-2. Short-cut method to determine the crystallite size at 90% conversion.

T (°C)	t_{90} (min)	*D* (nm) [a]
25	142	10.4
40	43	10.1
60	24	11.3
80	48	12.5
100	36	12.6
120	51	13.2

a. Using the equation *D* (nm)=*A*(*T*)·α(*t*), where the *A*(*T*) values are given in Figure S-3.

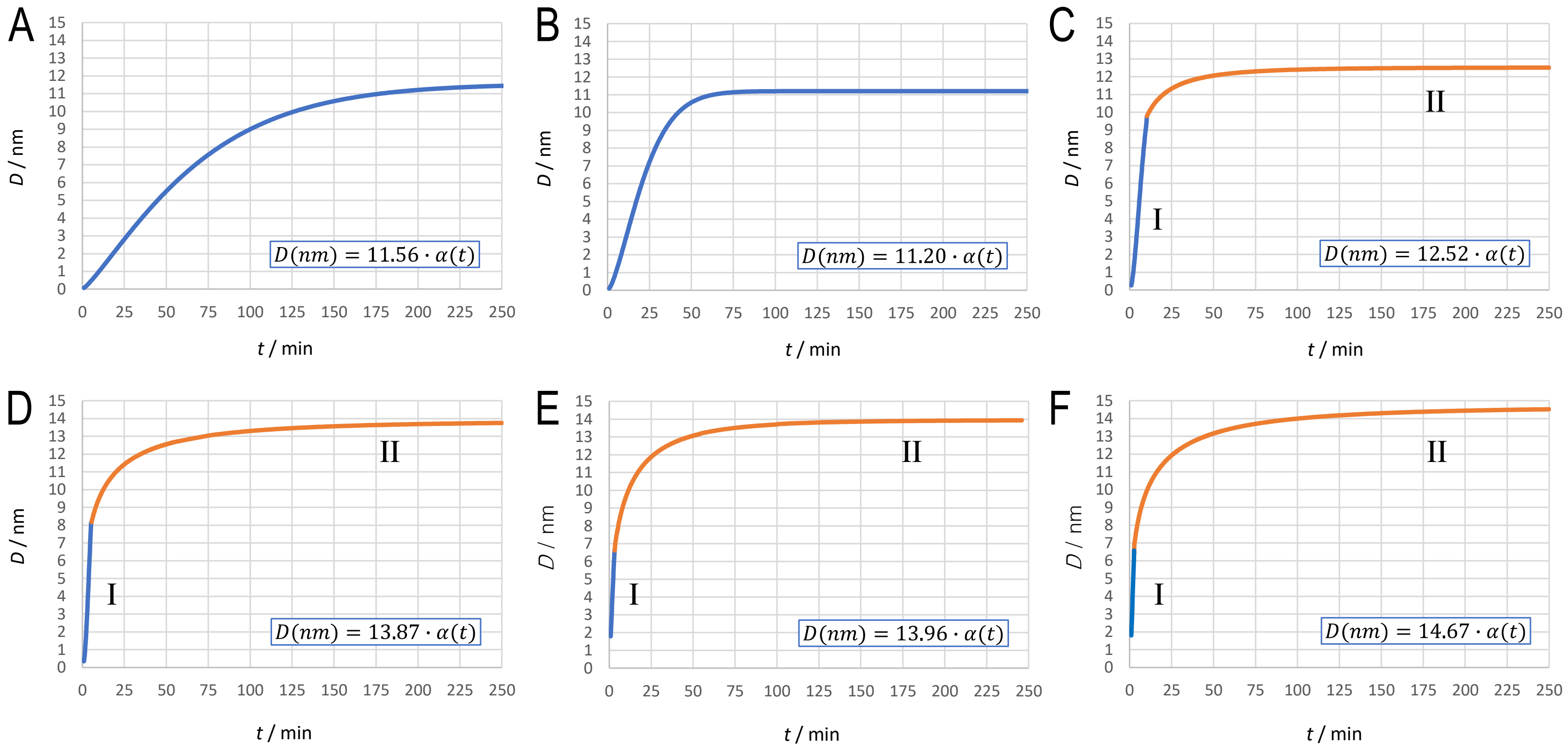

Figure S-3. Shortcut method to assess the evolution of the crystallite size based on a simplified model: D (nm)=$A(T)\cdot\alpha(t)$, where $A(T)$ is a parameter depending on temperature and a(t) is the conversion given by the Avrami-Erofe'ev model (Figure 2 of the main text). The parameter $A(T)$ was calculated from the average crystallite size reported by Millange *et al.* (*J. Mater. Chem*. **2000**, 10, 1713-1720) at 100 min reconstruction. The here-obtained equations are given on each graph. The model is simplified because the α-curves are based on the available intensity growth of the (003) hydrotalcite reflection, rather than the peak width (which is not available): **A**) 25 °C, **B**) 40 °C, **C**) 60 °C, **D**) 80 °C, **E**) 100 °C and **F**) 120 °C.
